# Effects of Workflow Optimization in Endovascularly Treated Stroke Patients – A Pre-Post Effectiveness Study

**DOI:** 10.1371/journal.pone.0169192

**Published:** 2016-12-30

**Authors:** Katharina Schregel, Daniel Behme, Ioannis Tsogkas, Michael Knauth, Ilko Maier, André Karch, Rafael Mikolajczyk, José Hinz, Jan Liman, Marios-Nikos Psychogios

**Affiliations:** 1 Department of Neuroradiology, University Medicine Goettingen, Goettingen, Germany; 2 Department of Neurology, University Medicine Goettingen, Goettingen, Germany; 3 Department of Infectiology, Helmholtz Center for Infection Research, Braunschweig, Germany; 4 Department of Anaesthesiology, University Medicine Goettingen, Goettingen, Germany; "INSERM", FRANCE

## Abstract

Endovascular treatment of acute ischemic stroke has become standard of care for patients with large artery occlusion. Early restoration of blood flow is crucial for a good clinical outcome. We introduced an interdisciplinary standard operating procedure (SOP) between neuroradiologists, neurologists and anesthesiologists in order to streamline patient management. This study analyzes the effect of optimized workflow on periprocedural timings and its potential influence on clinical outcome. Data were extracted from a prospectively maintained university hospital stroke database. The standard operating procedure was established in February 2014. Of the 368 acute stroke patients undergoing endovascular treatment between 2008 and 2015, 278 patients were treated prior to and 90 after process optimization. Outcome measures were periprocedural time intervals and residual functional impairment. After implementation of the SOP, time from symptom onset to reperfusion was significantly reduced (median 264 min prior and 211 min after SOP-introduction (IQR 228–32 min and 161–278 min, respectively); *P*<0.001). Especially faster supply of imaging and prompt transfer of patients to the angiography suite contributed to this effect. Time between hospital admission and groin puncture was reduced by half after process optimization (median 64 min after versus 121 min prior to SOP-introduction (IQR 54–77 min and 96–161 min, respectively); *P*<0.001). Clinical outcome was significantly better after workflow optimization as measured with the modified Rankin Scale (common odds ratio (OR) 0.56; 95% CI 0.32–0.98; *P* = 0.038). Optimization of workflow and interdisciplinary teamwork significantly improved the outcome of patients with acute ischemic stroke due to a significant reduction of in-hospital examination, transportation, imaging and treatment times.

## Introduction

Endovascular treatment (EVT) for acute ischemic stroke has developed significantly over the last decades and is now considered standard of care for patients with large artery occlusion (LAO) [1–5]. Amongst others, this development has been based on significant improvements of the technical aspects of EVT, which now allow for a swift and complete reperfusion in the majority of cases. Clinical outcome is not only dependent on successful EVT alone but also on several other factors. A short time from symptom onset to reperfusion is crucial for better clinical outcome and therefore any unnecessary delay to treatment has to be avoided. In this context and after publication of multiple negative trials in 2013 [6–8], we identified time from admission to reperfusion as a major issue in stroke treatment, that could be significantly improved in our hospital.

To streamline the “door to reperfusion” process, we established an interdisciplinary standard operating procedure (SOP) between neurologists, neuroradiologists and anesthesiologists aiming at shorter door to reperfusion times. This study analyzes the effects of an optimized workflow on periprocedural timings and its potential influence on clinical outcome after EVT in a pre-post comparison at a comprehensive stroke center.

## Materials and Methods

### Initial Situation

EVT of acute stroke patients with LAO is performed in our hospital since 2006. We started to register procedural timings, clinical data and interventional features in a comprehensive database in February 2008, which was approved by our local ethics committee (Ethikkommission der Universitaetsmedizin Goettingen, approval number 4/11/08 and 15/7/13). Patients’ consent for treatment was obtained according to common clinical guidelines. Our ethics committee waived the need for a separate consent concerning the inclusion in our observational database.

When a patient with symptoms suggestive of acute stroke was admitted to the hospital, he firstly was examined neurologically in the emergency room (ER). The neurologist in charge assessed the National Institutes of Health Stroke Scale (NIHSS), took blood samples and placed a large peripheral venous catheter. Then, the neuroradiologist was called and asked for imaging. Even though CT was the preferred imaging modality for acute stroke in our hospital, few patients were examined using MRI. The decision for one or the other modality was at the discretion of the neuroradiologist. The patient was transferred to the respective imaging facility and in most cases, nonenhanced CT as well as CT angiography (CTA) and CT perfusion (CTP) were performed. MRI examination included T2-weighted sequences, Time-of-Flight MR-angiography, diffusion weighted imaging and perfusion. After imaging and exclusion of an intracranial bleeding by the neuroradiologist, the neurologists administered recombinant tissue plasminogen activator (rtPA) if the patient was eligible. In several cases, the patient was transferred back to the ER for the administration of rtPA. Meanwhile, the interventional neuroradiologist was called, evaluated the images and discussed with the treating neurologist, whether an EVT should be performed. Patient selection for EVT was not standardized.

If so, the patient was transferred to the angiography suite and the anesthesiologist was informed. The anesthesiologist placed an arterial catheter for intraarterial blood pressure measurement, a urinary catheter and anesthetized the patient generally in the majority of cases. After that, the interventional neuroradiologist prepared the patient for EVT and started treatment.

### Standard Operating Procedure

**Fig 1:**
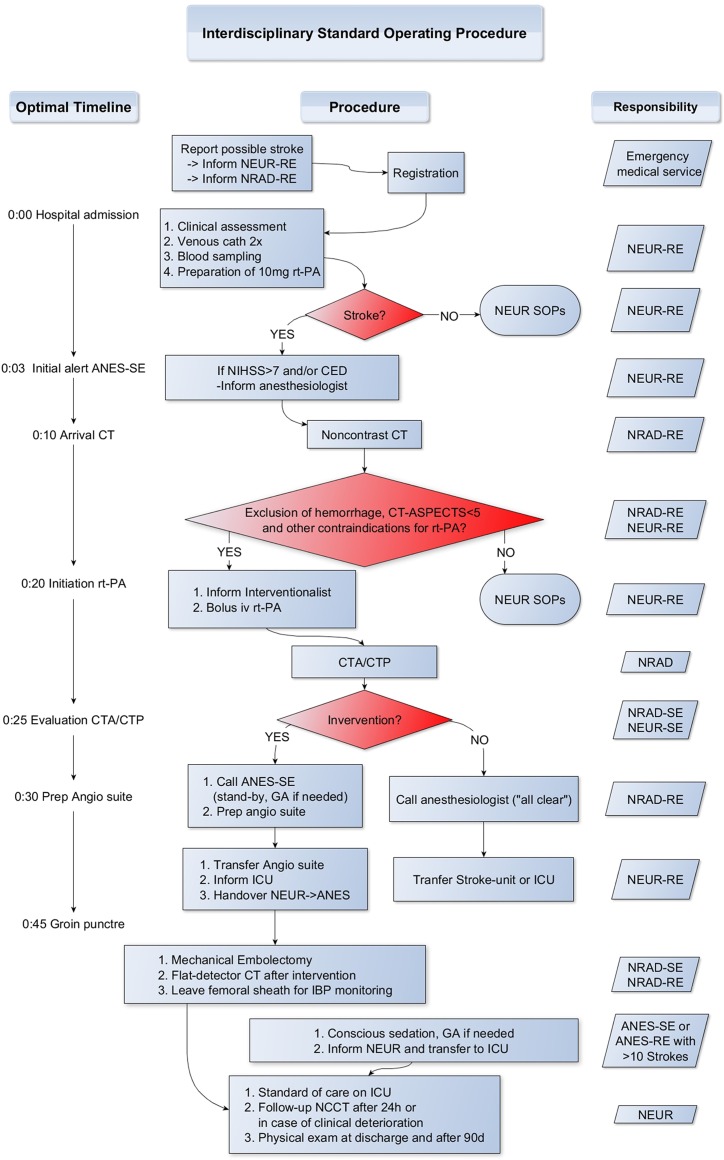
Flow chart of our interdisciplinary standard operating procedure. NEUR-RE, resident neurologist; NRAD-RE, resident neuroradiologist; rt-PA, recombinant tissue plasminogen activator; SOP, standard operating procedure; ANES-SE, senior anesthesiologist; NIHSS, national institute of health stroke score; CED, conjugate eye deviation; ASPECTS, alberta stroke program early CT score; CTA, CT angiography; CTP, CT perfusion; NRAD-SE, senior neuroradiologist; NEUR-SE, senior neurologist; GA, general anesthesia; ICU, intensive care unit; IBP, invasive blood pressure; ANES-RE, resident anesthesiologist; NCCT, noncontrast CT.

In February 2014, we developed and introduced an interdisciplinary SOP in collaboration with neurologists and anesthesiologists in order to optimize management of patients with acute stroke. We put emphasis on reducing times from patient admission to reperfusion. A flow chart depicting the intended workflow is shown in Fig 1.

As soon as the emergency medical service informs the neurological ER about a patient with signs suggesting an acute stroke, the neurologists informs the diagnostic neuroradiologist. After admission, a rapid clinical assessment is performed including quantification of functional impairment according to NIHSS. Meanwhile, a blood sample is taken and two large peripheral venous catheters are placed. If the neurologist still suspects an acute stroke, he escorts the patient to the imaging site taking a backpack containing emergency equipment plus a complete rtPA set including 90mg rtPA, necessary syringes, i.v. lines and a syringe pump. Target time for these procedures is 10 minutes. In case of a NIHSS value above 7 and/or conjugate eye deviation the anesthesiologist is informed (first call) that there might be an upcoming endovascular treatment. The imaging modality of choice is CT. Only if this is not possible or there are reasons favoring an MRI, the neuroradiologists can decide to perform MRI instead of CT. Noncontrast CT is performed immediately. The neuroradiologist reads the images quickly. After exclusion of an intracranial hemorrhage and an already demarcated large infarct, the neurologist administers rtPA intravenously while the patient lies on the CT table. Targeted “door to needle” time is 20 minutes. CTA and CTP are conducted subsequently. Meanwhile, the interventional neuroradiologist is called. Then, it is evaluated, whether the patient is eligible for EVT. This decision is based on infarct size and presence of LAO on CTA. As patients with large demarcated infarcts are not likely to benefit from treatment, we quantify infarct size using the Alberta Stroke Program Early CT score (ASPECTS) [9,10]. An infarct is considered as large if the ASPECTS value is below 5. Lack of opacification on CTA is considered as sign for artery occlusion. In summary, EVT is performed within the first 6 hours after symptom onset if the patient presents with following imaging criteria:

- ASPECTS ≥ 5 on nonenhanced CT
- presence of LAO on CTA

Patients with symptom duration of 6 to 12 hours are still eligible for EVT if they present with an ASPECTS of 5 or above and if there is still salvageable tissue. The latter is evaluated by applying ASPECTS to maps of the cerebral blood volume (CBV) gathered with CTP [11]. Cut-off for an EVT in the 6 to 12 hour time window is a CBV-ASPECTS of 6 and above.

This selection process should not take longer than 5 minutes. During this, the neurologist remains with the patient on-site. If the patient is eligible for EVT, the anesthesiologist is informed (second call) and the patient is directly transferred to the adjacent angiography suite. Primarily, the patient should be managed under conscious sedation. In cases where conscious sedation is not sufficient due to sustained agitation or movements, patients will be intubated for general anesthesia. To facilitate this, the angiographic table can be swayed towards the ventilation machine, so that no additional transfer of the patient is needed and anesthesiologists are not disturbed by the C-arm. The blood pressure is measured noninvasively, but closely monitored during the intervention. The anesthesiologist takes care to prevent hypotension during the procedure. Meanwhile, the neuroradiological team prepares the patient for EVT. All preparations should be done in parallel and taken within another 10 minutes as we are aiming for a total time of 45 minutes from patient admission to groin puncture (“door to puncture” time). After reperfusion, flat-panel angiographic CT is performed in order to rule out periprocedural complications (i.e. intracranial hemorrhage). Afterwards, the patient is transferred to ICU/Stroke Unit. A follow-up CT is performed after 24 hours or earlier in case of clinical deterioration. The patient is invited for a neurological follow-up after 90 days including evaluation of residual functional impairment according to the modified Rankin Scale (mRS) and a transcranial Doppler.

Regular interdisciplinary team meetings were established to train all neuroradiologists, technicians, neurologists, nurses and anesthesiologists for the new processes. Additionally, a training video was produced, which is permanently available for all staff involved and which is used to train new employees. Case conferences were held to discuss problems and potential solutions especially in the introduction stage, but are maintained on a regular basis. The actually achieved times are evaluated and compared to the target times every three months. Possible discrepancies are discussed in interdisciplinary team meetings and tried to be cleared out within the following period.

Table 1 compares the management of patients with acute stroke before and after implementation of the SOP.

**Table 1:** Comparison of workflows prior and after introduction of the SOP.

	Pre-SOP	Post-SOP
ER		information of neuroradiologist prior to arrival of patient with suspected stroke
	neurological assessment upon patients’ arrival, blood samples, i.v. catheter placement	rapid clinical assessement, blood samples, placement of 2 large i.v. catheters
	information of neuroradiologist	first call to anethesiologist
	transport to imaging facility	immediate transport to imaging facility
Imaging	NCCT + CTA + CTP	NCCT
	patient transfer back to ER for administration of rtPA; in some cases on-site administration	i.v. rtPA on-site, if patient eligible
	call interventional neuroradiologist	CTA + CTP, meanwhile call interventional neuroradiologist
	selection of patients eligible for EVT based on non-standardized assessment of interventional neuroradiologist and senior neurologist	standardized selection process for EVT
	patient transfer to angiography suite	if patient eligible, immediate transfer to angiography suite and second call to anesthesiologist
	call to anesthesiologist	
EVT	anesthesiologist places i.a. and urinary cathether and anesthetizes patient generally	neuroradiological team and ER-neurologist prepare patient for EVT and start EVT; anesthesiologist starts conscious sedation and prepares patients for general anesthesia if necessary
	neuroradiological team prepares patient for EVT	interventional neuroradiologist performs EVT
	interventional neuroradiologist performs EVT	

SOP, standard operating procedure; ER, emergency room; i.v., intravenous; NCCT, noncontrast CT; CTA, CT angiography; CTP, CT perfusion; EVT, endovascular treatment.

### Patient Population and Study Design

This study is a retrospective interpretation of a longitudinal prospectively maintained database approved by the local ethics committee. For this study, we screened our database for all patients with acute ischemic stroke who underwent EVT from 2008–2015. The SOP outlined above was introduced in February 2014. We identified a total number of 368 patients of whom 278 were treated before process optimization (February 2008 –January 2014) and 90 after introduction of the SOP (February 2014 –August 2015). The consulting neurologist assessed baseline NIHSS, mRS and cardiovascular risk factors directly after patient admission. A successful reperfusion was defined by modified thrombolysis in cerebral infarction score (mTICI) ≥2b [12] and absence of embolization to new vascular territory corresponding to recommendations of the cerebral angiographic revascularization group [13]. The evaluated time points were defined as follows: Symptom onset was considered as last time point the patient was or was seen well. Admission was the time when patient data were registered electronically in the hospital information system. The completion of the nonenhanced CT is regarded as imaging time. Needle time is the time point when intravenous administration of rtPA was started. Reperfusion time was specified as the time point of the first angiographic series depicting mTICI≥2b reperfusion.

Outcome measures were periprocedural time intervals and residual functional impairment before and after process optimization (February 2014). Firstly, time from symptom onset to admission, imaging, needle and reperfusion were calculated. Then, time from admission to imaging, needle, groin and reperfusion as well as time from imaging to groin and reperfusion were identified. Finally, groin to reperfusion time was determined. Analyses including reperfusion as time point correspond only to cases with successful reperfusion. Clinical outcome was evaluated according to the degree of functional impairment measured with mRS. In a final step, we analyzed the devices used for EVT in order to see whether use of retrievable stents had an influence on procedural duration.

### Statistical Analyses

Demographic characteristics of patients before and after implementation of SOP were compared with Mann-Whitney-U and Fisher’s exact tests. Time to event analyses were performed using multivariable Cox regression models to determine if time periods differed before and after introduction of the SOP. A hazard ratio (HR) above 1 indicated that the time interval was shorter after process optimization. In order to evaluate if discharge mRS values differed before and after implementation of the SOP ordered logistic regression was performed. Here, an odds ratio below 1 indicated that the mRS values after process optimization were lower. We additionally investigated, if outcome measures changed over time based on a learning effect of the treatment team. We addressed this potential bias by including calendar time as an additional predictor in analyses stratified by time before and after introduction of the SOP.

All multivariable analyses were adjusted for potential confounders. This included a priori confounders like age, sex, baseline mRS and CT- as well as CBV-ASPECTS. Variables with baseline imbalances were assessed for a potential confounding effect on the analyses of interest and were included in multivariable models as appropriate. Results were classified as significant when *P* value was < 0.05.

Statistical analyses were performed using MedCalc (MedCalc Software version 14, Ostend, Belgium) and Stata 12 (StataCorp, College Station, TX, USA).

## Results

Baseline characteristics and scores before and after implementation of the SOP are shown in Table 2.

**Table 2:** Characteristics of patients prior and after implementation of the SOP.

	Total (n = 368)	Prior to SOP (n = 278)	After SOP (n = 90)	*P*
Age, median (IQR), y	72 (60–79)	71(60–78)	76 (62–80)	0.049
**Medical comorbidities**				
Hyperlipidemia	196 (55%)	165 (60%)	31 (37%)	<0.001
Hypertension	297 (82%)	229 (82%)	68 (79%)	0.524
Diabetes mellitus	90 (25%)	69 (25%)	21 (25%)	1
Smoking	70 (35%)	48 (41%)	22 (26%)	0.036
PAD	25 (7%)	20 (7%)	5 (6%)	1
Obesity	126 (45%)	108 (55%)	18 (21%)	<0.001
**Clinical scores, median (IQR)**				
Admission NIHSS, median (IQR)	16 (11–21)	17 (11–22)	16 (11–20)	0.204
Admission mRS, median (IQR)	5 (4–5)	5 (4–5)	5 (4–5)	0.306
Discharge NIHSS, median (IQR)	9 (3–17)	9 (4–18)	7 (2–15)	0.043
Discharge mRS, median (IQR)	4 (2–5)	4 (2–5)	4 (1.5–5)	0.085
**Imaging scores, median (IQR)**				
CT ASPECTS	8 (7–9)	8 (7–9)	8 (7–9)	0.883
CTA ASPECTS	7 (5–8)	7 (5–8)	7 (6–8)	0.247
CBV ASPECTS	7 (5–8)	6 (5–8)	7 (6–8)	0.011
Successful Reperfusion (mTICI≥2b)	191 (55%)	132 (51%)	59 (66%)	0.019
**Occlusion sites**				0.316
proximal ICA	11 (3%)	10 (4%)	1 (1%)	
Carotid-T	51 (14%)	43 (16%)	8 (9%)	
M1	206 (56%)	151 (54%)	55 (61%)	
M2	27 (7%)	18 (6%)	9 (10%)	
BA	71 (19%)	54 (19%)	17 (19%)	
PCA	2 (1%)	2 (1%)	0	
**Administration of rtPA**	244 (66%)	176 (64%)	68 (75,6%)	0.053

SOP, standard operating procedure; IQR, interquartile range; PAD, peripheral artery disease; NIHSS, National Institutes of Health stroke scale; mRS, modified Rankin score; ASPECTS, Alberta stroke program early CT scale; CTA, CT angiography; CBV, cerebral blood volume; mTICI, modified thrombolysis in cerebral infarction; ICA, internal carotid artery; M1, M1-segment of the middle cerebral artery; M2, M2-segment of the middle cerebral artery; BA, basilary artery; PCA, posterior cerebral artery.

Patients after SOP implementation were older (*P* = 0.049), leaner (*P*<0.001), less likely to smoke (*P* = 0.036) or suffer from hyperlipidemia (*P*<0.001) and showed higher CBV ASPECTS scores (*P* = 0.011). Accordingly, we adjusted all further analyses for these variables with baseline imbalances and assessed if they changed the effect estimates any further. This was not the case.

Time from symptom onset to admission did not differ significantly before and after introduction of the SOP (median 77 minutes and 80 minutes; HR 1.02; 95% confidence interval (CI) 0.79–1.32; *P* = 0.882, see Table 3).

**Table 3:** Treatment times of patients prior and after implementation of the SOP.

	Total (n = 368)	Prior to SOP (n = 278)	After SOP (n = 90)	*P*	*HR (95% CI)*
Onset to admission, median (IQR), minutes	77 (52–130)	77 (52–132)	80 (51–126)	0.882	1.02 (0.79–1.32)
Onset to mTICI2b, median (IQR), minutes	253 (202–310)	264 (228–327)	211 (161–278)	<0.001	1.76 (1.36–2.28)
Onset to needle, median (IQR), minutes	110 (105–122)	125 (93–169)	105 (80–138)	0.043	1.27 (0.84–1.82)
Admission to imaging, median (IQR), minutes	26 (18–39)	31 (22–46)	19 (13–24)	<0.001	2.12 (1.64–2.74)
Admission to needle, median (IQR), minutes	31 (22–41)	34 (27–54)	26 (20–35)	0.017	1.59 (1.09–2.31)
Admission to groin, median (IQR), minutes	107 (70–141)	121 (96–161)	64 (54–77)	<0.001	3.65 (2.82–4.73)
Imaging to groin, median (IQR), minutes	72 (48–112)	85 (60–125)	43 (35–55)	<0.001	3.20 (2.48–4.14)
Groin to mTICI≥2b, median (IQR), minutes	52 (36–77)	58 (39–84)	42 (30–60)	<0.001	1.45 (1.12–1.87)

All analyses were adjusted for age, sex, baseline mRS and CT- as well as CBV-ASPECTS; adjusting for smoking, obesity and hyperlipidemia did not change effect estimates for SOP any further. SOP, standard operating procedure; HR, hazard ratio; CI, confidence interval; IQR, interquartile range; mTICI, modified thrombolysis in cerebral infarction.

Additionally, times from symptom onset to imaging and to administration of rtPA remained similar (HR 1.05; 95% CI 0.81–1.35; *P* = 0.732 and HR 1.27; 95% CI 0.84–1.82; *P* = 0.180 respectively).

Only few patients of each group underwent MRI (4,5% prior to and 5,4% after introduction of the SOP). As the percentages were similar, we did not adjust for the use of MRI in further analyses.

The total time between symptom onset and reperfusion was reduced significantly (median 264 minutes before and 211 minutes after SOP; HR 1.76; 95% CI 1.36–2.28; *P*<0.001). Especially the faster supply of imaging (HR 2.12; 95% CI 1.64–2.72, *P*<0.001) as well as the prompt transfer of patients to the angiography suite (HR 3.20; 95% CI 2.48–4.14, *P*<0.001) contributed to this effect. The duration between hospital admission and groin puncture as well as the time from imaging to groin puncture were reduced by half after implementation of the new SOP (median 121 and 85 minutes before; 64 and 43 minutes after implementation of the new SOP; HR 3.65 and 3.2; 95% CI 2.82–4.73 and 2.48–4.14; *P*<0.001 for both). EVT was performed under general anesthesia in the vast majority of patients (94,3%) prior to the implementation of the SOP. In contrast, we were able to treat one-fifth of the patients (21,2%) under conscious sedation after introduction of the SOP. The remaining (78,8%) had to be intubated for general anesthesia during the intervention.

The duration of endovascular treatment was shorter after introduction of the SOP (HR 1.45; 95% CI 1.12–1.87; *P* = 0.005). We evaluated whether the used devices accounted for this. We found that the time reduction was independent from the use of retrievable stents (HR 1.03; 95% CI 0.80–1.33, *P* = 0.814). Consequently, admission to reperfusion and imaging to reperfusion times were reduced after applying the new workflow (median 175 and 147 minutes before, 105 and 87 minutes with the new workflow; HR 2.32 and 2.65; 95% CI 1.80–2.99 and 2.02–3.47; *P*<0.001 for both).

**Fig 2:**
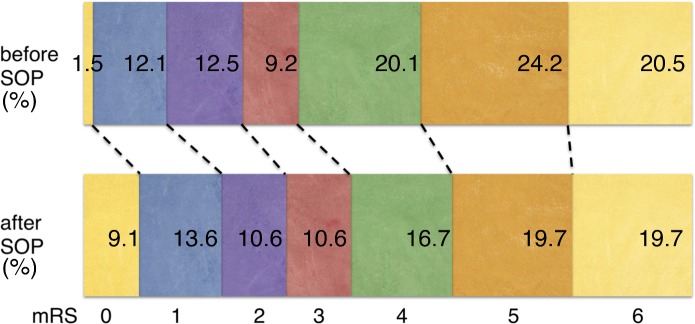
Bar graph of patient outcome measured with modified Rankin scale (mRS). Colors represent the scores ranging from 0 (no symptoms at all) to 6 (dead). Percentages of each category are given. After introduction of the standard operating procedure there were significantly more patients with no residual functional impairment. A general shift towards lower mRS values can be noticed (depicted with dotted lines). Hence, after management optimization less patients remained severely disabled.

When focusing on clinical outcome of endovascularly treated stroke patients, a significant decrease of mRS values could be shown after optimization of workflow (OR 0.56; 95% CI 0.32–0.98; *P* = 0.038). There were not only more patients with no residual functional impairment (mRS = 0; 1.5% before and 9.1% after workflow optimization) but also less severely disabled patients (mRS = 4–5; 44.3% and 36.4% respectively) after introduction of the SOP as shown in Fig 2.

The effect was even stronger among patients with successful reperfusion (OR 0.36; 95% CI 0.17–0.75; *P* = 0.006). Implementation of the new SOP had no effect on clinical outcome in cases of ineffective reperfusion (OR 1.58; 95% CI 0.58–4.29; *P* = 0.367). Overall, we report higher reperfusion rates after optimizing workflow (66% vs. 51%, *P* = 0.019) and better clinical outcome in patients with successful reperfusion. However, the effect on clinical outcome was perceivable only five months after implementation of the SOP (OR 1.03; 95% CI 0.39–2.70; *P* = 0.942). In the following, an impact of the SOP on clinical outcome was observable and remained constant over time (OR = 0.44; 95% CI 0.23–0.83; *P* = 0.01).

We found that calendar time was not associated with outcome before introduction of the SOP (*P* = 0.283). This also held true for length of treatment.

## Discussion

In this study, we showed that optimized management of patients with acute stroke and increased awareness of medical staff related to their treatment reduces times from symptom onset to reperfusion significantly. Given the time-dependent nature of ischemic pathology [14], time to reperfusion plays a critical role regarding clinical outcome [15]. All influenceable delays of treatment should be avoided [16]^,^[17]. Consequently, we demonstrated that clinical outcome of patients was significantly better after introduction of our interdisciplinary SOP. Our analyses ruled out, that improved outcome originated from mere learning of the treatment team. Calendar time was not associated with outcome measures prior to introduction of the SOP, indicating that there was no training effect. Outcome measures remained unaltered in the first five months with the SOP. This can be attributed to a preparatory period needed to implement the protocol and to train all staff involved. Regular team meetings and case conferences helped to solve problems and to establish a high awareness concerning the procedures. This was reflected in a considerable improvement of outcome after the first five months.

As expected, times from symptom onset to hospital admission remained similar before and after introduction of the SOP. This period can hardly be influenced by the treating stroke physicians, as it depends on many patient-inherent factors like recognition of symptoms, seeking for help and availability of medical care close to the place of residence. Our primary aim therefore was to reduce times from patient admission to reperfusion as this period is mainly influenced by in-hospital factors. We achieved this by process acceleration and optimization of interdisciplinary cooperation. Faster supply of imaging and accelerated transportation of the patient to the imaging and the angiography suite respectively contributed primarily to the timesaving.

We tried to comply with requested target times in the literature. Goyal et al. demanded an imaging to reperfusion time of less than 60 minutes [18] and discouraged the use of CTP, in order to further reduce imaging times. Contrary to this opinion and findings from other authors [18,19], we showed that perfusion imaging does not lead to a significant delay of treatment. The median duration between imaging and groin puncture was 43 minutes (interquartile range 37–60). This period is even shorter as described in the “Endovascular Treatment for Small Core And Anterior Circulation Proximal Occlusion With Emphasis On Minimizing CT to Recanalization Times” (ESCAPE) trial, where the median process time was 51 minutes (interquartile range 39–68) [20], even though CTP was not acquired in this study. Furthermore, our total time from symptom-onset to reperfusion was 215 minutes on average. Thereby we are largely in the requested time frame of 300 minutes, where there is high probability of a good clinical outcome after successful reperfusion [21,22]. Additionally, a recent meta-analysis of the five randomized trials that demonstrated the benefit of EVT performed by the “Highly Effective Reperfusion Evaluated in Multiple Endovascular Stroke Trials (HERMES)” collaboration demonstrated that increasing delays of treatment were associated with higher levels of disability among patients treated endovascularly [23].

We standardized image evaluation in order to deliver EVT to eligible patients as fast as possible. We utilized ASPECTS for the quantification of early ischemic changes and infarct size. Only patients with an ASPECTS value above or equal to 5 were admitted to EVT as the benefit from intra-arterial treatment progressively decreases with declining values [24–27]. We optionally evaluated ASPECTS on CTP-maps in patients with prolonged symptom duration as described above. A previous study showed that perfusion parameters analyzed according to ASPECTS are more sensitive and specific than CT-ASPECTS [28]. Thus, we decided that patients with symptom duration of six to twelve hours and a CBV-ASPECTS value of 6 or above should still be treated endovasculary.

The establishment of the SOP allowed for a remarkable halving of the time between admission and groin puncture. Important in this context is amongst others, that conscious sedation is preferentially used. It is not only faster, but seems to be favorable for outcome as well, as it bears a lower risk for hypotension. Intraprocedural hypotension is an independent predictor for poor clinical outcome in endovasculary treated patients [29] and should therefore be avoided. Even though the majority of patient needed to be put under general anesthesia during EVT, we favor the use of initial conscious sedation. As groin to reperfusion time was significantly shorter after implementation of the SOP, a short interruption of EVT for intubation does not seem to have a significant retarding effect. One could argue, that the improved outcome of patients after introduction of the SOP is not only based on optimization of workflow but mainly due to faster and more successful reperfusion by using advanced thrombectomy devices like retrievable stents. In our analyses this hypothesis does not hold true. Firstly, duration of EVT was moderately shorter after introduction of the SOP. Secondly, there was no statistical evidence that application of retrievable stents accelerated the total time to reperfusion.

Although we successfully accelerated the management of patients with acute ischemic stroke, additional improvements are conceivable. Direct transportation of stroke patients to the CT scanner can further reduce door to imaging times. The combination of imaging and treatment in the angio suite could decrease potential in-hospital time delays even more [30]. Soderman et al. showed that reconstructions of CT from flat-panel detector images yield satisfactory results compared to conventional images [31]. Flat-panel CT has been shown to be of equal diagnostic value as multidetector CT for the detection of intracranial hemorrhage [32]. Another study suggested that measurements of CBF and CBV are feasible with flat-panel detector angiographic systems [33]. Therefore, we have recently started transporting drip and ship patients with LAO, who are referred to us from primary stroke centers, directly to our angio suite (bypassing ER and CT). There, we rapidly start the interventional procedure after ruling out interim hemorrhage with nonenhanced flat-panel CT.

The main limitations of our study are the retrospective analysis of prospectively acquired data and the relatively small sample size, which can mainly be attributed to the single-center design. Secondly, raters of reperfusion rates and neurological outcome measures were not blinded regarding patient groups.

In conclusion, interdisciplinary team work and implementation of a new SOP led to a significant reduction of in-hospital examination, transportation, imaging and treatment times in our stroke center. Better clinical outcomes were reported with faster in-hospital times, heightened awareness and improved interdisciplinary collaboration.
